# A Semi-Empirical Deflection-Based Method for Crack Width Prediction in Accelerated Construction of Steel Fibrous High-Performance Composite Small Box Girder

**DOI:** 10.3390/ma12060964

**Published:** 2019-03-22

**Authors:** Bishnu Gupt Gautam, Yi-Qiang Xiang, Zheng Qiu, Shu-Hai Guo

**Affiliations:** College of Civil Engineering and Architecture, Zhejiang University, Hangzhou 310058, China; bishnu@zju.edu.cn (B.G.G.); qiuzheng_zju@126.com (Z.Q.); 15158016702@163.com (S.-H.G.)

**Keywords:** static behavior, studs, deflection, finite element model, composite beam, crack width

## Abstract

Accelerated construction in the form of steel–concrete composite beams is among the most efficient methods to construct highway bridges. One of the main problems with this type of composite structures, which has not yet been fully clarified in the case of continuous beam, is the crack zone initiation that gradually expands through the beam width. In the current study, a semi-empirical model was proposed to predict the size of cracks in terms of small box girder deflection and intensity of load applied on the structure. To this end, a set of steel–concrete composite small box girders were constructed by the use of steel fibrous concrete and experimentally tested under different caseloads. The results were then used to create a dataset of the box girder response in terms of beam deflection and crack width. The obtained dataset was then utilized to develop a simplified formula providing the maximum width of cracks. The results showed that the cracks initiated in the hogging moment region when the load exceeded 80 kN. Additionally, it was observed that the maximum cracked zone occurred in the center of the beam due to the maximum negative moment. Moreover, the crack width of the box girder at different loading cases was compared with the test results obtained from the literature. A good agreement has been found between the proposed model and experiment results.

## 1. Introduction

Advancement in new construction techniques has received much attention in the last decades. The conventional bridge constructions and rehabilitation are usually accompanied by several operational difficulties. In order to overcome the traditional bridge construction problems, quite a few construction techniques were established such as accelerated, rapid, modular, mechanized, or precast constructions. Among them, the accelerated construction is an effective technique to reduce the impact of the bridge construction period on the surrounding traffic flow [1]. Moreover, it can properly help the engineering projects to save the implementation cost, improve the construction safety, reduce the adverse effects on the surrounding environment, ensure the higher construction quality, etc. [2].

**Figure 1:**
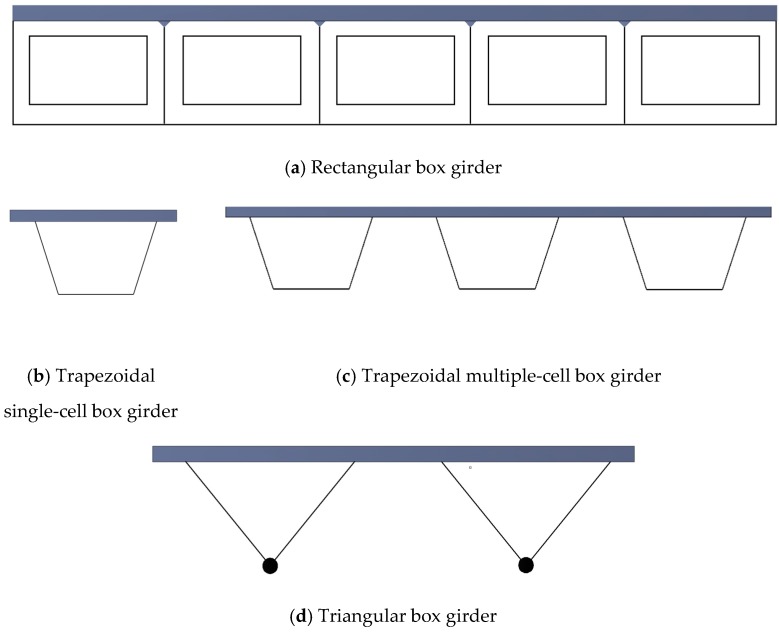
Different cross-sections of box girders: (**a**) Rectangular box girder; (**b**) trapezoidal single-cell box girder; (**c**) trapezoidal multiple-cell box girder; (**d**) triangular box girder.

Accelerated construction of steel fibrous high-performance continuous composite box girder bridge (ACHPCBG-bridge) is classified into two types—steel–concrete composite box girder bridge with single or double cell and small box girder bridge with multiple cells [3]. If the depth of the box girder exceeds 1/6 or 1/5 of the bridge width, single-cell box girder is recommended to be used in practice; however, if the depth is smaller than 1/6 of the width of bridge, double-cell or multiple-cell box girders can be considered [4]. The boxes used in the ACHPCBG-bridge are mostly rectangular, trapezoidal, or triangular in shape [5,6] as shown in Figure 1. It is reported that triangular boxes with apex down suffer from some disadvantages [7]. They usually have to be deeper than rectangular or trapezoidal boxes. Additionally, because of smaller area, triangular boxes have less torsional resistance. Furthermore, their bottom flange often has to be a heavy built-up section combined with bent plates for connection to the webs. Bending moment and shear force on rectangular box girders are greater than that of trapezoidal cross section [8]. Therefore, trapezoidal box girders may be a better choice for accelerated construction in comparison to other box girders. Characteristics of concrete material is also an important part in steel–concrete composite box girders, especially for cases where a large strength-to-weight ratio is required [9]. A box girder is either simply supported or continuous. Many researchers simulated the composite structures in bridge application to study various aspects of their structural behaviors [10,11,12,13,14,15,16,17]. 

The ultimate loading capacity of the ACHPCBG-bridge is usually determined by either flexural or shear bearing capacity and is governed by the compressive strength of the concrete or tensile strength of the steel girder. Moreover, in some areas with a continuous composite beam subjected to hogging moment, high strength is required to resist the negative bending. In the support region where the negative bending moment acts, a relatively high tensile stress is generated in a concrete slab and compressive stresses are induced in the lower steel region. As such, the mechanical behavior of these girders is strongly nonlinear even for low-level stress at that region, resulting in crack initiation in the slab, which is generally considered as a shortcoming for the durability and service life of a structure. [18,19,20,21,22,23]. 

In any construction, the characteristics of concrete play an essential role. Concrete is brittle and has limited ductile behavior. Therefore, a form of reinforcement is required to enhance structural stability. Steel bars are used as reinforcement in concrete structures; however, there is still a possibility of crack formation internally or externally, which may lead to a major problem in overall stability of structure [24,25].

The cracks developed in reinforced concrete members extend freely until encountering a reinforcing bar. We need to arrest the cracks to lengthen the lifespan of structures. Hence, a multi-directional and closely spaced reinforcement is required to be used in concrete. The fibers are, thus, an excellent choice to overcome this type of problem in practice. Fiber reinforced concrete (FRC) is composed of short discrete fibers that are uniformly distributed and randomly oriented within the concrete matrix. The fibers are mainly classified into four types—steel, glass, natural, and synthetic—and each of the different fibers have different properties [26,27,28,29,30].

Fibers in a concrete element increase the structural integrity, provide high tensile strength to plain concrete, reduce the permeability of concrete, and increase the resistance to impact load. Fibers can reduce the number of rebars without loss of strength. They can also eliminate cracks propagation by bridging action. The flexural behavior, bond strength, and especially toughness of SFRC increase as the fiber content increases. Carbon or steel fibers can be added to a cement matrix at a high volume fraction (0.5–3%) to increase the conductivity of the composite. The properties of fiber concrete depend upon the volume of fibers used [31,32,33,34,35,36].

Various types of problems occur in composite materials, mostly due to cracks and delamination. The crack in a composite structure may reduce the structural stiffness and strength and redistribute the load, which may either delay the structural failure or accelerate the structural collapse. The crack is not the main cause of structural failure, but rather is the part of the failure process that may lead to the loss of structural integrity. Therefore, it plays an important role in the failure mechanism of steel–concrete composite structures [37,38,39,40,41,42,43].

The composite beam usually consists of a steel section jointly acting with one (or two) flange(s) made of reinforced concrete that is mainly subjected to bending [44]. These two materials are interconnected by means of mechanical shear connectors. Therefore, a composite beam, even with small steel sections, has high stiffness and carries heavy loads on long spans. However, by increasing the load intensity, additional issues such as slip and deflection occur along the beam [45]. In the current study, different deformation gauges were installed at the bottom of flanges of the beam to measure the maximum deflection induced along the beam.

The current study mainly aimed at investigating the static behavior of the ACHPCBG-bridge under vertical loading and proposing a simplified methodology to estimate the width of cracks. For this purpose, an ACHPCBG-bridge was experimentally tested under several caseloads to get deflection response. Additionally, the cracking process at hogging moment region was measured at each load step. The details of experimental models are discussed in the following sections.

## 2. Design and Fabrication of Experimental ACHPCBG-bridge

Accelerated construction steel–concrete composite small box girder is a construction technique consisting of an open steel box girder, diaphragm, welding stud, and concrete slab. It is difficult to make corresponding scale models, especially of the concrete bridge deck and the thickness of the steel plate. Therefore, based on the 25 m span prototype bridge, a stereotype model of the steel–concrete composite small box girder was prepared with the members-to-actual length ratio of 1:4. The basic parameters of box girder are shown in Table 1. 

In the accelerated construction of steel–concrete composite small box girder, concrete material, stud group arrangement, and stud group spacing are three main parameters. At present, ordinary concrete by a grade size below C50 is mainly used in steel–concrete composite structures. However, because of the high strength and low weight, high-performance concrete not only reduces the weight of superstructure and decrease the costs, but also provides higher durability to the main structure. Hence, it is gradually getting more applications in bridge engineering. As to the knowledge of the authors, there is no specific literature or standard available to give unified understanding of the arrangement and spacing of studs in group. From the design point of view, in order to ensure the mechanical performance of the bridge structure, the degree of shear connection should not be too small; however, from the viewpoint of construction process, it is desirable to increase the stud spacing as much as possible to adapt the accelerated construction. The current code for design of steel–concrete composite structures in China [46] requires that the number of welding studs in each shear span area should be greater than the ratio of longitudinal shear force at the interface between steel beam and concrete slab and the shear capacity of single welding stud, i.e., complete shear design is required. In the steel structure design code [47], when the strength and deformation are satisfied, the longitudinal and horizontal shear capacity of the shear connectors at the interface of composite beams can guarantee the full flexural capacity of the maximum moment section; then, they can be designed according to the partial shear connection. However, partial shear connections are limited to composite beams with equal cross-section spans not exceeding 20 m. The AASHTO [48] bridge design code stipulates that the spacing of reserved hole should not be more than 610 mm. EC4 [49] requires that the maximum spacing of uniform welding studs should not exceed the minimum of 4 times the thickness of concrete slab and 800 mm; however, there is no specific provision for group stud shear connectors. 

**Figure 2:**
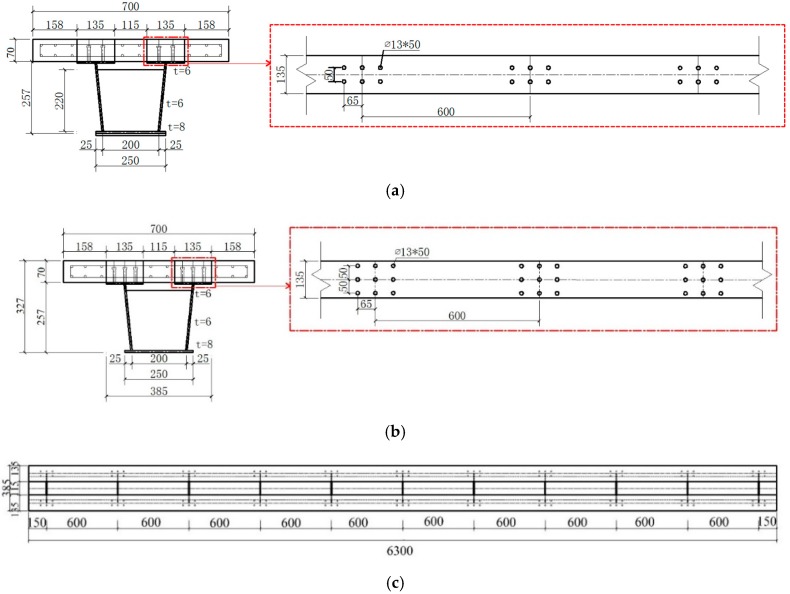

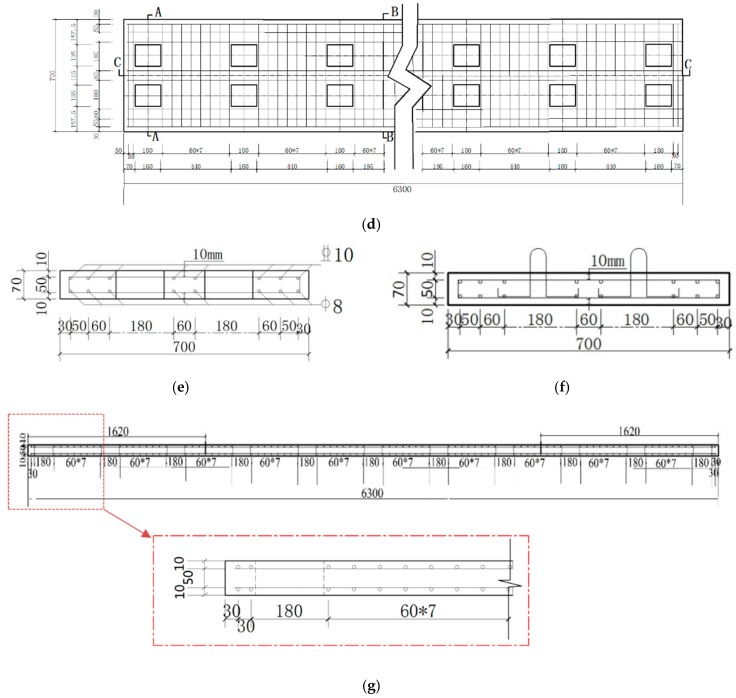
Details of experimental accelerated construction of steel fibrous high-performance continuous composite box girder bridge (ACHPCBG-bridge) (mm). (**a**) Composite beam cross-section and stud arrangement of specimen 1; (**b**) composite beam cross-section and stud arrangement of specimen 2; (**c**) plan of steel; (**d**) plan of concrete; (**e**) cross-section at A-A; (**f**) cross-section at B-B; (**g**) cross-section at C-C.

**Figure 3:**
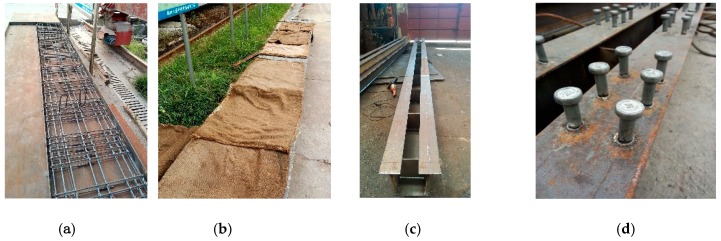

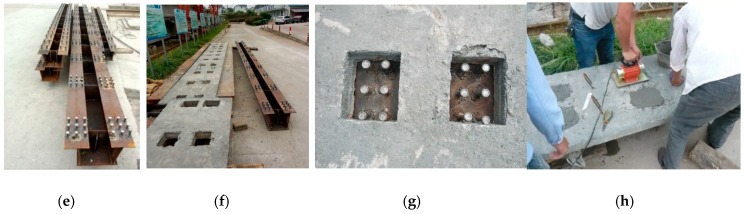
Fabrication process of specimen: (**a**) Formwork erection and reinforcing cage binding; (**b**) casting and maintenance of concrete slab; (**c**) steel beam welding; (**d**) stud welding of test specimen 1; (**e**) stud welding of test specimen 2; (**f**) completion of concrete slab and open steel box girder; (**g**) placing of concrete slab on steel box girder to make it composite; (**h**) reserve hole filling.

The structure and main dimension of the composite test beam are shown in Figure 2, which is made up of an open steel box girder and precast concrete slab. The test beam was 6.3 m in total length. It had two spans, with each span of 3 m length. The total height of the composite box girder was 327 mm with 70 mm slab thickness and 257 mm height of steel box girder. The upper flange was 6 mm thick and 135 mm wide. The web and bottom plates were 6 mm and 8 mm thick, respectively. A solid web diaphragm was set at every 600 mm from the support position. The diaphragm was 6 mm thick and 220 mm high. The test beam was made of Q345qc steel. The upper flange of the steel beam was arranged with shear group studs. The center distance of the group stud was 600 mm. The lateral distance of the group stud was 50 mm and the longitudinal distance was 65 mm. The welding stud was Φ13 × 50 made of ML15AL.The configuration of the steel–concrete composite beam is given in Figure 2. Construction of prefabricated concrete slabs, welding of steel beams, and welding of studs are strictly in accordance with the requirements of design, drawings, and construction technique specifications. The main fabrication process is shown in Figure 3. Copper-plated steel fiber with a diameter 0.2 mm and length of 13 mm, tensile strength of 2000 MPa, and volume fraction of 1.5% was used in a concrete element. The physical properties of steel fiber used are shown in Appendix A, Table A3. The information about different properties and materials used, such as cement, fine aggregates, coarse aggregate, water, and chemicals have been described in details in Appendix A.

## 3. Experimental Test

### 3.1. Test Method and Instrumentation

#### 3.1.1. Deflection

**Figure 4:**
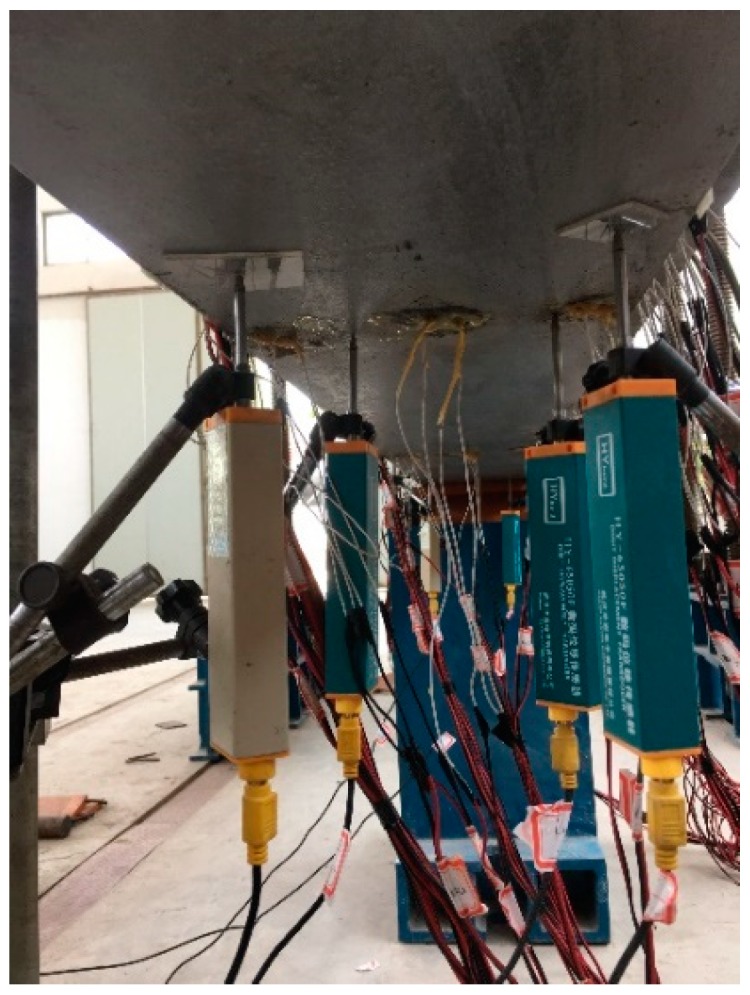
The arrangement of the displacement sensor.

Deflection was measured by means of digital displacement transducer deformation gauges at key sections as shown in Figure 4. At the key sections, two sensors were used on each side of the steel bottom to get the accurate measurement of displacement. Due to limitation of instrument available in the lab, a set of two actuators with a total imposing force of 900 kN was applied to the model. The numerical model was established as a first approximation in order to find the experimental result properly. The deflection at 900 kN was experimentally found as 13.211 mm, which is very close to the analytical value of 12.8 2 mm. The details about obtained results from the experimental test and numerical analysis is presented in the establishment of numerical modeling section.

#### 3.1.2. Crack Measure

The digital concrete crack gauges were used to measure the crack width at a key section (i.e., the negative bending moment region) of the concrete slab. This crack gauge was used for quantitative detection of the crack width on the concrete surface, known as the common concrete non-destructive testing equipment. The gauge could automatically interpret the measurement and directly display the crack width value on the screen. It had a measuring range of 0.01 to 3 mm, the magnification of 60×, and the estimated accuracy of 0.005. At different loading cases, crack length and crack width were measured. The observed maximum crack width was considered for formulation and verification. In the experimental test of specimen 1, crack number 3 had the maximum crack width of 0.456 mm at the 900 kN load; for specimen 2, however, crack number 2 had the maximum crack width of 0.349 mm. The detail of crack width, crack length, and loading cases of specimens 1 and 2 are presented in Table 2 and Table 3, respectively. More details about crack formation is discussed in Section 6, simplified model.

### 3.2. Loading Procedure

**Figure 5:**
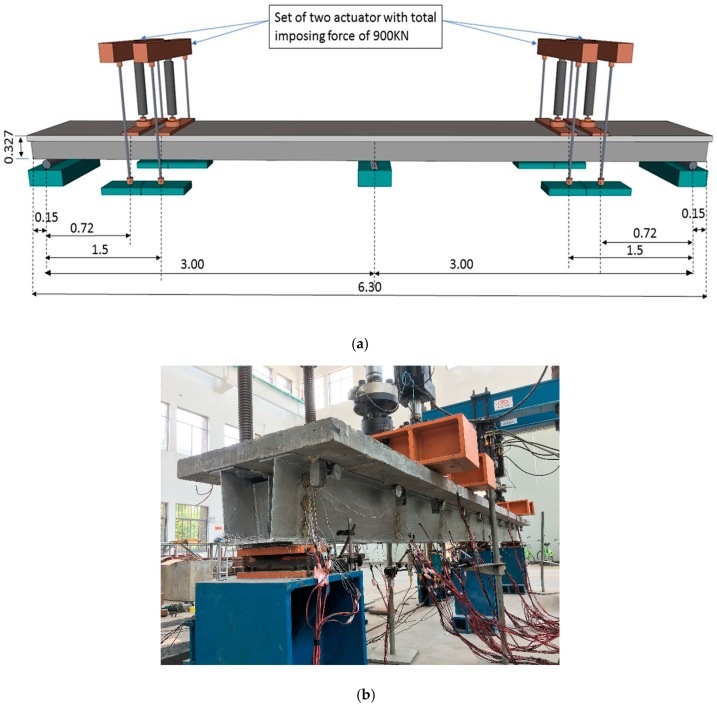
The typical geometry and dimensions of the test specimen: (**a**) Schematic front view (m); (**b**) side view.

The experiment was performed in the structural lab of the Quzhou University. A set of two actuators with total 900 kN loading capacity equipment was used in the experiment to apply the load at the mid-span of the steel–concrete composite beam. The test specimen was supported by a roller system at both ends and hinge support at the center of the two-span continuous beam. The change in behavior of composite girders was carefully observed through the static test. The test girder was loaded up to 100 kN in 20 kN intervals, followed by 50 kN intervals up to 900 kN. The maximum capacity of the instrument was 900 kN. A loading plate (110 cm × 30 cm × 20 cm) and a supporting plate (70 cm × 15 cm × 8 mm) have been used in the experiment. The test setup for the steel–concrete composite beam is illustrated in Figure 5

## 4. Establishment of Numerical Modeling

### 4.1. Modeling

**Figure 6:**
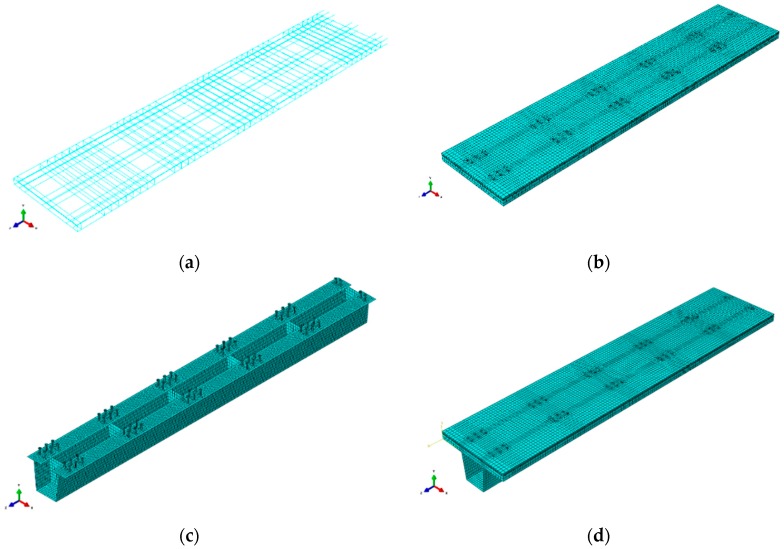

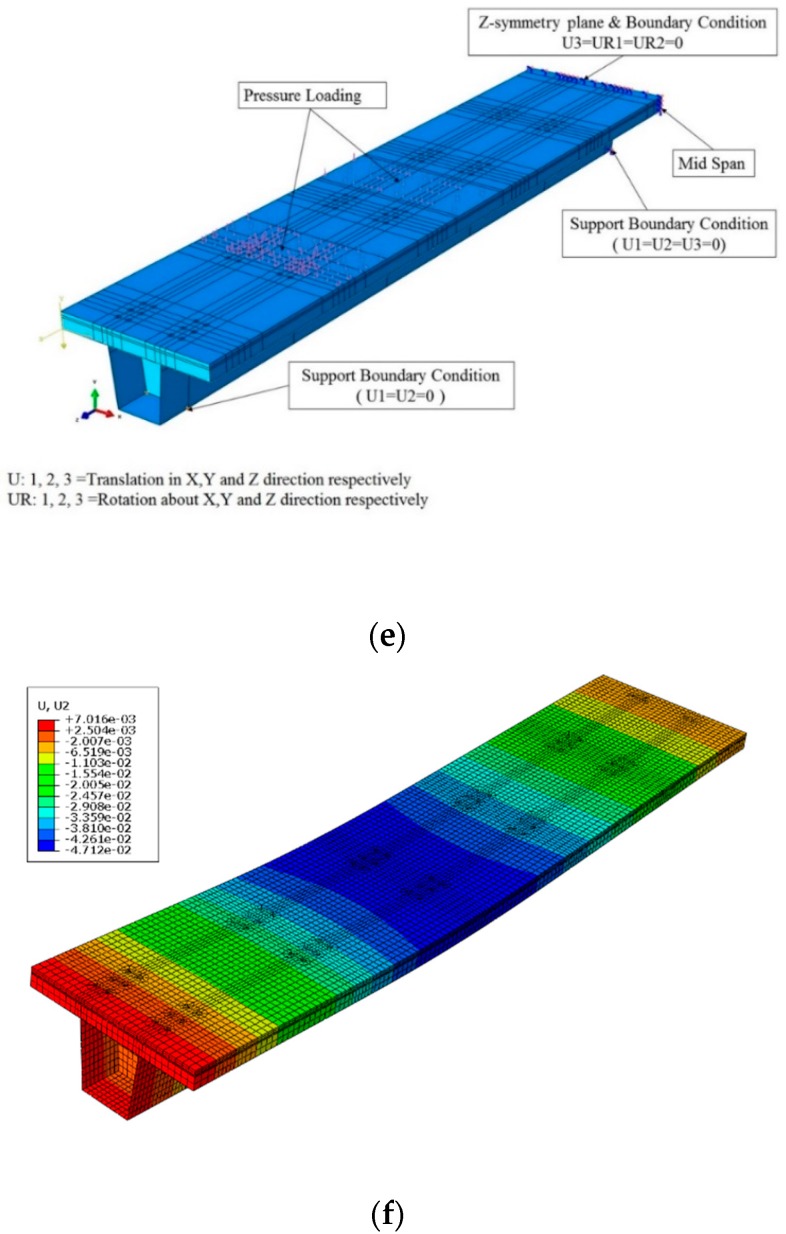
The finite element model: (**a**) Reinforced-steel meshing; (**b**) concrete-slab meshing; (**c**) steel beam, diaphragm, and stud meshing; (**d**) composite box girder meshing; (**e**) boundary conditions; (**f**) load-deflection behavior at 1000 kN load.

The specimen had two spans of 3 m and the total length of the girder was 6.3 m. A 3D numerical model of the steel–concrete composite girder beam with a clear span of 3 m was simulated in ABAQUS version 6.14 [50]. A detailed view of the numerical model is shown in Figure 6. Loading was applied to the top surface of the concrete slab and distributed over the full width of the girder. The load increased with 20 kN intervals up to 100 kN and, then, the load interval was set to 50 kN up to the total imposing force of the actuator. Due to the symmetry in geometry, loading, and boundary conditions, only half of the beam was modeled. The coordinate system represented axes X, Y, and Z as axes 1, 2, and 3 in the model, respectively. The symmetry boundary conditions are shown in Figure 6e with a restrained degree of freedom [51].

ABAQUS/standard solver with a linear geometric order was used in the study. Eight-node brick elements with reduced integration (C3D8R) were used to model the concrete and stud. The first-order interpolation, three-dimensional beam element (B31), and four-node shell element with reduced integration (S4R) were used to model reinforcement bars and steel, respectively. The final mesh included 55,496 elements and 67,327 nodes. Steel reinforcement bars were modeled using embedded rebar element. Surface-to-surface contact was used for stud-to-concrete and steel-to-concrete interactions. The material nonlinearities of concrete and steel were modeled using the concrete damage plasticity model and the elastic-plastic bilinear model, respectively. The accuracy of the results basically depends on the size of the mesh, the constitutive model, and boundary conditions [52]. Therefore, these aspects should be carefully incorporated into the proposed finite element model. Adequate attention was paid to the development of hexahedral mesh and the assigning interaction between various surfaces. Various components, namely, concrete slab, steel beam, stud connectors, reinforcement bars, and stiffeners, were meshed part by part instead of using global or sweep features. Thus, a regular structured hexahedral mesh was generated. To get an acceptable level of accuracy, the approximate global mesh size of 0.025 was used for reinforcement bars and concrete; whereas an approximate global mesh size of 0.015 was used for studs and steels. The modeling of different parts of the beam is shown in Figure 6. Material and geometrical details of the specimen girder are given in Table 1.

### 4.2. Material Description

#### 4.2.1. Concrete

In order to simulate the mechanical behavior of concrete, a damage plastic model of concrete was utilized in the current study. The constitutive relationship of concrete is adopted from the uniaxial tension and compressive stress–strain curve of concrete given in the “code for the design of concrete structure” GB 50010-2010 [53]. On the day of the experiment, the measured average compressive strength of C60 concrete cube was 76.24 MPa, and the design strength of reserved-hole C80 concrete was 87.4 MPa. Additionally, the average splitting strength and the average elastic modulus were 5.09 MPa and 36.4 GPa, respectively. The uniaxial tension and the uniaxial compression parameters are listed in Table 4.

The stress–strain relationship of concrete under uniaxial tension is expressed as follows:

(1) $$\sigma =\left(1-d_{t}\right)E_{c}\varepsilon$$

(2) $$d_{t}=1-\rho_{t}\left[1.2-0.2x^{5}\right],\;x\leq 1$$

(3) $$d_{t}=1-\frac{\rho_{t}}{\alpha_{t}{\left(x-1\right)}^{1.7}+x},\;x>1$$

(4) $$x=\frac{\varepsilon }{\varepsilon_{t,r}}$$

(5) $$\rho_{t}=\frac{f_{t,r}}{E_{c}\varepsilon_{t,r}}$$

where $\alpha_{t}$ is the parameter of concrete uniaxial tension stress–strain curve in the decline period, $f_{t,r}$ is the representation of concrete uniaxial tensile strength, $\varepsilon_{t,r}$ is the peak tensile strain corresponding to $f_{t,r}$, and $d_{t}$ is the evolution parameter of concrete under uniaxial tension.

Compression stress–strain relationships are assumed as follows:

(6) $$\sigma =\left(1-d_{c}\right)E_{c}\varepsilon$$

(7) $$d_{c}=1-\frac{\rho_{c}n}{n-1+x^{n}},\;x\leq 1$$

(8) $$d_{c}=1-\frac{\rho_{c}}{\alpha_{c}{\left(x-1\right)}^{2}+x},\;x>1$$

(9) $$\rho_{c}=\frac{f_{c,r}}{E_{c}\varepsilon_{c,r}}$$

(10) $$n=\frac{E_{c}\varepsilon_{c,r}}{E_{c}\varepsilon_{c,r}-f_{c,r}}$$

(11) $$x=\frac{\varepsilon }{\varepsilon_{c,r}}$$

where $\alpha_{c}$ is the parameter of concrete uniaxial compression stress–strain curve in the decline period, $f_{c,r}$ is the representation of concrete uniaxial compressive strength, $\varepsilon_{t,r}$ is the peak compressive strain corresponding to $f_{c,r}$, and $d_{c}$ is the evolution parameter of concrete under uniaxial compression.

**Figure 7:**
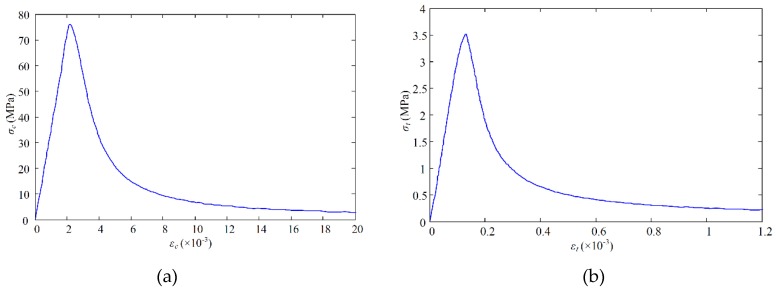
The stress–strain curve of C60 Concrete. (**a**) Compression; (**b**) tension.

The stress–strain relationship and damage model of C60 concrete are shown in Figure 7.

#### 4.2.2. Steel Beam, Stiffener, and Stud

**Figure 8:**
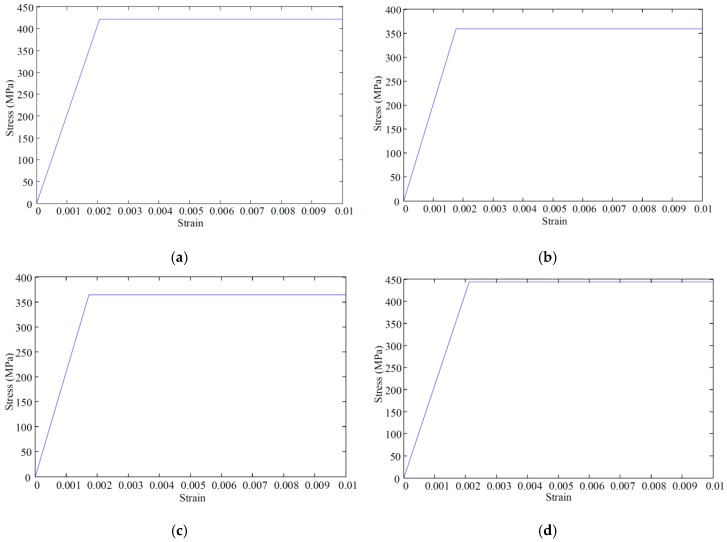
Stress–strain relationships: (**a**) The stress–strain curve of steel; (**b**) the stress–strain curve of stud; (**c**) the stress–strain curve of ɸ8 rebar; (**d**) the stress–strain curve of ɸ10 rebar.

The steel and stiffener were modeled considering the nonlinear behavior of the materials. The elastic-plastic material model was employed based on the nominal stress–strain behavior of steel. In the stud connection, an elastic-plastic bilinear model was utilized. However, the material of steel plates, studs, and steel bars was defined by the ideal elastoplastic model. That is, when the steel yields, the bearing capacity does not increase, but the deformation continues to increase. The stress–strain relationships of steel plates, studs, and bars are shown in Figure 8.

### 4.3. Bonding

The bonding between the materials was done by the use of interaction in Abaqus. The stud and concrete was modeled by the penalty method considering a friction coefficient of 0.4 in the tangential direction and hard contact in the normal direction to avoid penetration between the two contact surfaces [51]. On account of the interaction between the flange of steel and concrete slabs, the steel was determined as the “slave surface” and the concrete as the “master surface”. The finite sliding method was employed for the interaction between studs and concrete. To simulate the steel bar–concrete interaction, the reinforcement bar was selected as the embedded region and concrete was set to be the host region. 

### 4.4. Comparison Between Numerical Analysis Values and Experimental Results

The validation of experimental results was performed using the numerical analysis data as described earlier in Section 4. There was a good agreement between numerical and experimental results. Due to the maximum capacity of instrument available in the lab, a set of two actuators with a total imposing load of 900 kN was applied to the model. However, in the numerical model, the final step of the model was set to 1000 kN with the full shear interaction. The load increment was considered 10% in each load step. The numerical results showed a relatively stiffer behavior compared to the experimental model. It could be due to the stabilization process that occurred during the loading stage because of the change in loading system from one set of actuators to another set of actuators. However, similar results were obtained at the 900 kN load. Afterward, by a small increment in load, a significant change was observed in the deflection that resulted from the numerical model. Due to the symmetry in the geometric shape of the beam, the experimental measurement was performed in just one span. The deflection at 900 kN was analytically found as 12.82 mm, which is very close to the experimental value of 13.211 mm. The deflection obtained from the numerical analysis is presented in Table 5. 

## 5. Simplified Model

**Figure 9:**
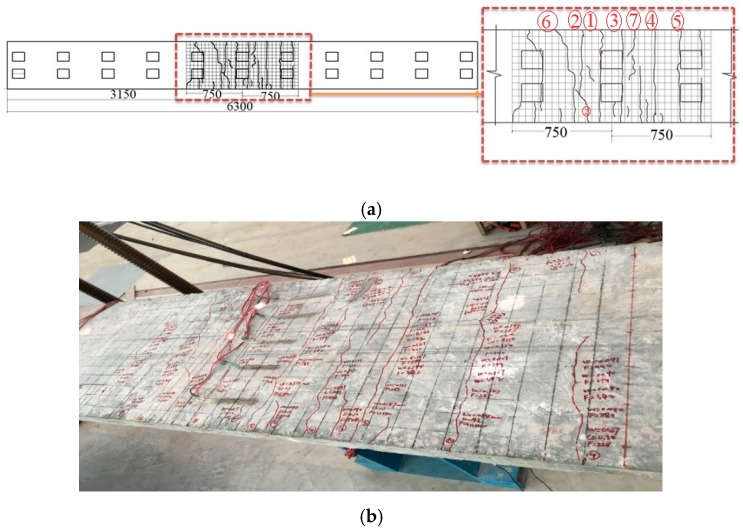
Formation and distribution of cracks in specimen 1: (**a**) Schematic view of cracks; (**b**) actual view of cracks.

**Figure 10:**
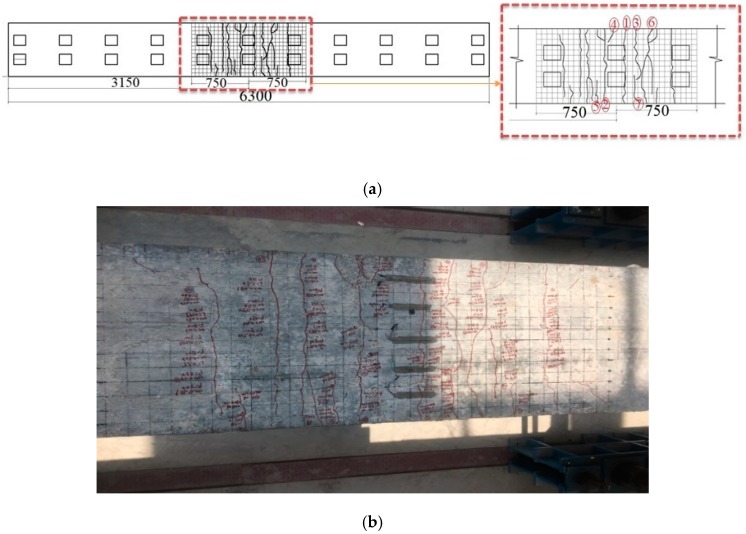
Formation and distribution of cracks in specimen 2: (**a**) Schematic view of cracks; (**b**) actual view of cracks.

The first crack observed when the load exceeded 80 kN; then, by increasing the load intensity, further crack formations were observed in the specimen. The cracks formed on the box girder were marked by a set of numbers demonstrating the occurrence order. Crack number 3 had the maximum crack width and was considered in the formulation process. The major cracks observed in the specimen are presented in Figure 9 and Figure 10. Additionally, the related data are listed in Table 2 and Table 3. According to EC4 [49], the effective length of the beam was considered 1500 mm. To observe the crack width and length and measure them accurately, a 5 × 5 cm grid size was made on concrete slab as demonstrated in Figure 9 and Figure 10. The relationship between crack width and maximum central deflection of the ACHPCBG-bridge is addressed in this section. This relationship relies on the evaluation of load-deflection behaviors and load-crack width behaviors of experimental model outcomes.

There are many situations where an individual wants to use a simplified model and finds a formula that best fits a given set of data. Simplifying the model is the best solution and is the process of constructing a mathematical function with the best fit to a series of data points, giving a mathematical ideal solution. 

**Figure 11:**
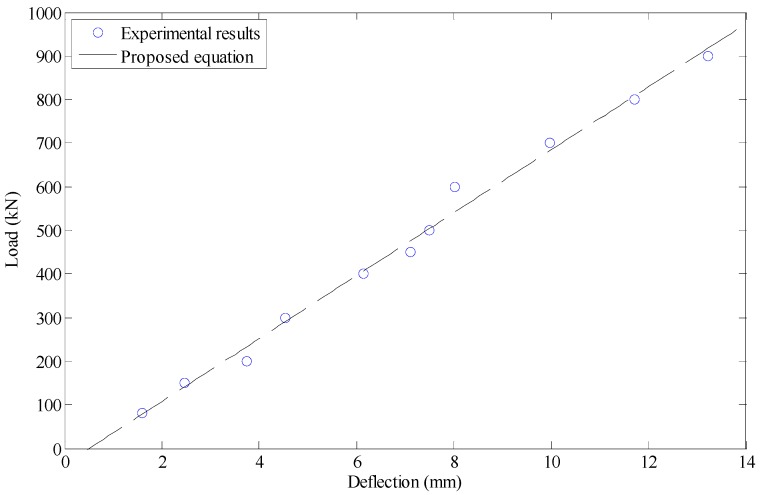
Fitting the load–deflection curve.

As the first step, with experimental results a simplified formula was developed between the deflection and the load applied on the beam. It was done by fitting a first-order polynomial function to the data presented in Figure 11. The result was as follows:

(12) L=72.183d−36.781

where d (m) is the deflection measured at upper yield point of elastic stage in the center of the beam and *L* (kN) is the intensity of the load excreted on the beam.

**Figure 12:**
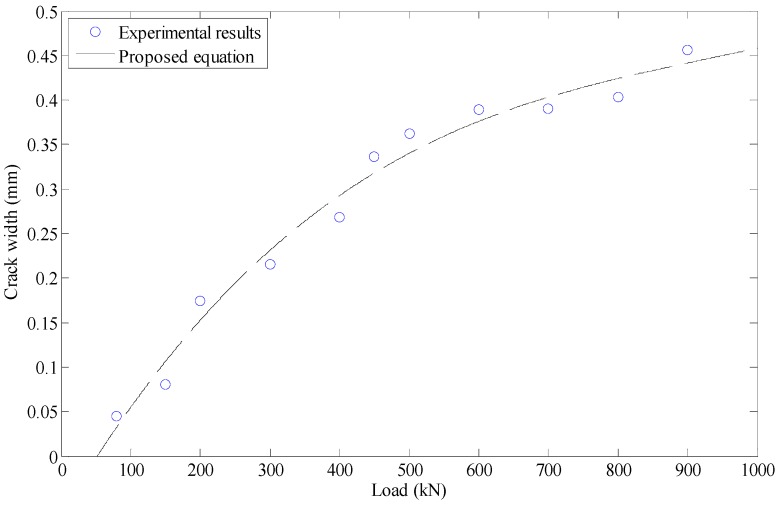
Fitting the load–crack width curve.

Next, using the data obtained from the experimental tests, a third-order polynomial regression model was developed between the load and crack width. It was done by fitting a third-order polynomial function to the data presented in Figure 12. The result was as follows:

(13) $$C=4*10^{-10}L^{3}-9*10^{-7}L^{2}+0.001L-0.0317$$

where *C* (mm) is the maximum width of crack observed before failure. Substituting Equation (12) in Equation (13) gives a new expression, as follows:

(14) $$C=1.5*10^{-4}d^{3}-4.9193*10^{-3}d^{2}+0.07707d-0.06971$$

**Figure 13:**
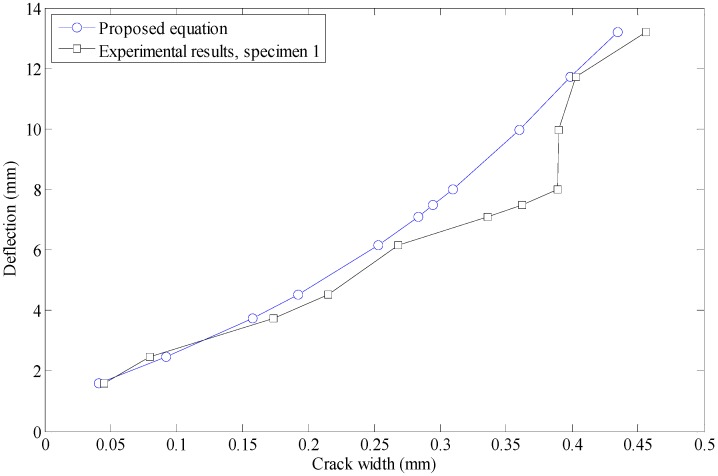
Crack width-deflection comparison of beam.

To adopt the proposed model for the prediction of crack size in steel–concrete composite small box girder, one can simply calculate the maximum deflection under the static load case. The equivalent intensity of the load can then be calculated using Equation (12). Next, Equation (13) can be utilized to approximate the maximum width of the crack generated in box girder beam. However, the limitation of the proposed model regarding the dimension and construction size should also be considered while utilizing the proposed model. Having Equation (14), the crack width is available at any desired beam deflection. The behavior of central deflection and crack width at negative bending moment region is shown in Figure 13.

## 6. Discussion

**Figure 14:**
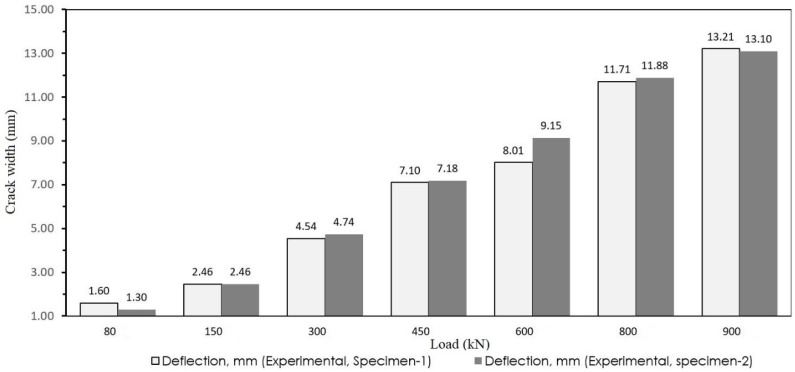
Load deflection comparison of tested specimens.

It is noteworthy that the provided relationship is limited to the upper yield points of the elastic stage. In order to validate the results and evaluate the accuracy of the proposed formula, a laboratory model was created. The experimental model was performed under different loads, and the maximum deflection was calculated in each case. Additionally, the crack width was measured from the experimental test. The results are reported in Table 6 and Table 7. The load-deflation comparisons of tested specimen are shown in Figure 14. Furthermore, the crack width was approximated by the proposed model for each load intensity and compared with the experimental results of specimen 1 and 2. 

**Figure 15:**
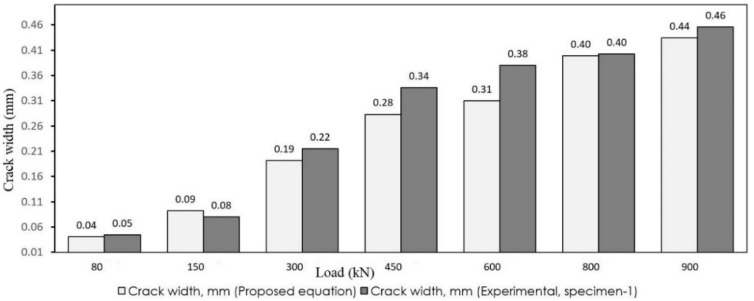
Comparison of crack width (specimen 1).

**Figure 16:**
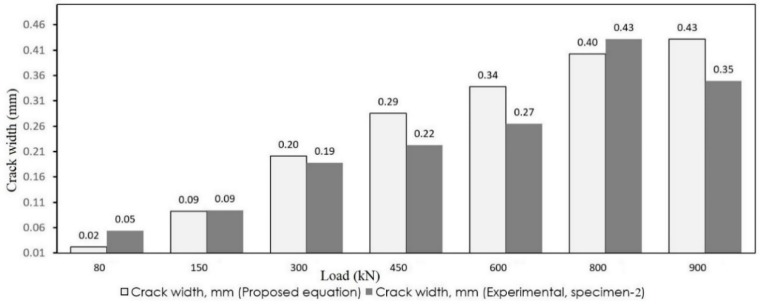
Comparison of crack width (specimen 2).

As observed, the crack widths obtained from the simplified model are appropriately close to the results of the experiments. To better observe the accuracy, the results of the proposed and experimental models are depicted in Figure 15 and Figure 16. The white bars show the crack width that resulted from Equation (14) and the gray bars depict the experimental results.

**Figure 17:**
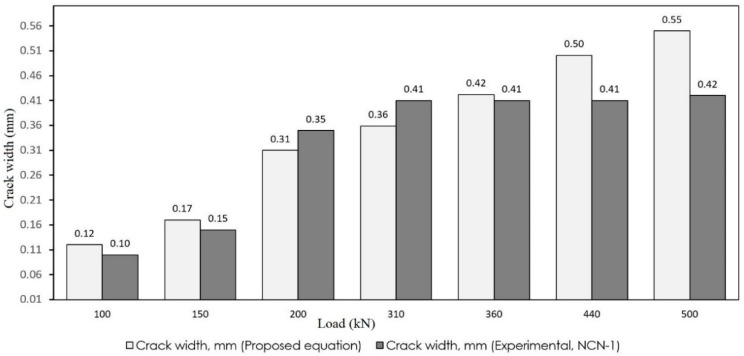
Comparison of crack width resulted from the proposed formula and experiments for the NCN-1 sample in Su et al.’s study [54].

**Figure 18:**
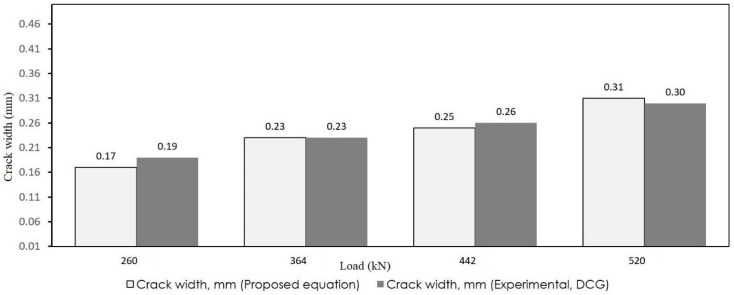
Comparison of crack width resulted from the proposed formula and experiments for the DCG sample in Xu et al.’s study [12].

To further evaluate the model, a few studies were selected from the literature to compare with the proposed formula. Su et al. [54] experimentally analyzed two different types of continuous composite box girders. One specimen was a conventional composite box girder with cast in situ (specimen NCN-1) slab whereas the other was a composite box girder with a prefabricated prestressed concrete slab (specimen NCN-2). Due to using prestressed concrete, the proposed formula yielded slightly higher results than the experimental results regarding NCN-2; however, in the case of NCN-1, the results of the proposed formula were in good agreement with the experimental results as shown in Figure 17. According to their research, no great increase of the crack width was found when load increased from 312 to 700 kN; but, in this stage, there was substantial increase in the amount of cracking. This stage is the stabilization process of the crack, which means the crack distribution experiences a transition from its randomly distributed state to a quasi-uniformly distributed state. Similarly, Xu et al. [12] manufactured and tested a continuous double composite girder (DCG) to study the mechanical behavior in negative flexural region. Their comparison results are shown in Figure 18.

## 7. Conclusions

The current study aimed at investigating the behavior of ACHPCBG-bridge utilizing experimental models. Therefore, a vertical loading was gradually applied to the beam, and the maximum deflection along the beam was observed at certain points. Additionally, the cracking mechanism was investigated by the experimental model and the maximum width of the cracks were measured by a digital crack gauge on the beam surfaces. Finally, a simplified formula was developed approximating the crack width as a function of deflection

The main conclusions drawn from the current study are as follows:

▪ A semi-empirical formula was developed based on experimental studies to approximate the width of crack in ACHPCBG-bridge.
▪ It was observed that the crack zone initiated when the load exceeded 80 kN with a crack width of 0.045 mm. The appeared crack propagated in full width, i.e., 0.7 m of the beam, when the load reached 150 kN. In this condition, the crack width was recorded as 0.08 mm.
▪ By the use of steel fiber in a concrete, the integrity and load resistance capacity are consequently increased. In this case, the maximum width of the crack was limited to 0.456 mm at 900 kN load.
▪ If construction can be done properly in a systematic manner, crack propagation would be effectively limited due to the bridging action of fibers.

Since the proposed formula is presented as an explicit function, it can be practically used to predict crack width. The proposed formula can also be used as a limit state function in reliability analysis to calculate the probability of failure for ACHPCBG-bridge. As the experiment models were designed by a 1:4 ratio based on 25 m prototype model, the application of the proposed method is limited to the models within a similar range of parameters. This issue can be investigated by the authors in future research.

## Figures and Tables

**Table 1:** Material and geometrical details of specimen girder.

Material	Parameter	Value	Parameter	Value
Concrete	Density, (kg/m^3^)	2400	Combined beam width, (mm)	700
	Elastic modulus, (MPa)	36,400	Beam length (calculated span), (cm)	630(600)
	Poisson’s ratio	0.2	Beam height, (mm)	327
	Yield strength, (MPa)	76.24	Number of welding studs	132
Steel	Density, (kg/m^3^)	7850	Design strength of bridge deck concrete, (MPa)	C60
	Elastic modulus, (GPa)	210	Thickness of concrete deck, (mm)	70
	Poisson’s ratio	0.3	Reserved hole concrete design strength, (MPa)	C80
	Yield strength, (MPa)	421	Spacing center distance, (mm)	600
Reinforcement	Density, (kg/m^3^)	7800	-	-
	Elastic modulus, (GPa)	206	-	-
	Poisson’s ratio	0.3	-	-
	Yield strength, (MPa)	445	-	-
Stud	Density, (kg/m^3^)	7800	-	-
	Elastic modulus, (GPa)	210	-	-
	Poisson’s ratio	0.3	-	-
	Yield strength, (MPa)	360	-	-
	Size, (mm)	Φ13 × 50	-	-

**Table 2:** Observed experimental results of specimen 1 (Unit: Crack length, m; crack width, mm).

Set	80 kN		150 kN		300 kN		450 kN		600 kN		800 kN		900 kN	
Crack number	Crack length	Crack width	Crack length	Crack width	Crack length	Crack width	Crack length	Crack width	Crack length	Crack width	Crack length	Crack width	Crack length	Crack width
1	0.052	0.041	0.7	0.063	0.7	0.134	0.7	0.174	0.7	0.221	0.7	0.309	0.7	0.309
2	0.049	0.04	0.7	0.092	0.7	0.148	0.7	0.215	0.7	0.228	0.7	0.228	0.7	0.228
3	0.058	0.045	0.7	0.08	0.7	0.215	0.7	0.336	0.7	0.389	0.7	0.403	0.7	0.456
4	-	-	0.7	0.064	0.7	0.121	0.7	0.161	0.7	0.186	0.7	0.228	0.7	0.228
5	-	-	-	-	0.42	0.107	0.42	0.107	0.7	0.134	0.7	0.161	0.7	0.161
6	-	-	-	-	0.45	0.127	0.45	0.161	0.53	0.174	0.53	0.188	0.53	0.188
7	-	-	-	-	0.11	0.181	0.11	0.181	0.11	0.181	0.11	0.181	0.11	0.181

**Table 3:** Observed experimental results of specimen 2 (Unit: crack length, m; crack width, mm).

Set	80 kN		150 kN		300 kN		450 kN		600 kN		800 kN		900 kN	
Crack number	Crack length	Crack width	Crack length	Crack width	Crack length	Crack width	Crack length	Crack width	Crack length	Crack width	Crack length	Crack width	Crack length	Crack width
1	0.7	0.054	0.7	0.107	0.7	0.174	0.7	0.161	0.7	0.255	0.7	0.295	0.7	0.322
2	0.58	0.054	0.7	0.094	0.7	0.188	0.7	0.223	0.7	0.265	0.7	0.336	0.7	0.349
3	-	-	-	-	0.13	0.054	0.17	0.067	0.44	0.121	0.7	0.188	0.7	0.215
4	-	-	-	-	0.12	0.094	0.11	0.107	0.11	0.134	0.12	0.188	0.12	0.188
5	-	-	-	-	0.21	0.054	0.21	0.054	0.21	0.067	0.21	0.107	0.21	0.094
6	-	-	-	-	0.57	0.094	0.7	0.134	0.7	0.188	0.7	0.215	0.7	0.242
7	-	-	-	-	0.18	0.054	0.21	0.094	0.22	0.107	0.22	0.134	0.7	0.134

**Table 4:** Concrete uniaxial tension and compression parameters.

Parameter	*α_t_*	*f_t,r_* (Mpa)	*ε_t,r_*	*α_c_*	*f_c,r_* (Mpa)	*ε_c,r_*
C60	3.82	3.5	1.28 × 10^−4^	3.81	76.24	2.19 × 10^−3^

**Table 5:** Load-deflection comparison of beam.

Load, kN	80	150	200	300	400	450	500	600	700	800	900	950	1000
**Deflection, FEM (mm)**	1.048	1.932	2.563	3.825	5.102	5.745	6.169	7.053	8.041	9.451	12.82	20.41	47.12
**Deflection Specimen 1 (mm)**	1.6	2.456	3.739	4.535	6.1465	7.1025	7.4925	8.0125	9.977	11.71	13.211	-	-

**Table 6:** Validation of the proposed relationship for crack width prediction of specimen 1.

Load, KN	Deflection, mm Experiment	Crack Width, mm (Experimental model)	Crack width, mm (Proposed formula)
80	1.6	0.045	0.041
150	2.456	0.08	0.092
300	4.535	0.215	0.192
450	7.102	0.336	0.283
600	8.012	0.389	0.31
800	11.71	0.403	0.399
900	13.211	0.456	0.435

**Table 7:** Validation of the proposed relationship for crack width prediction of specimen 2.

Load, KN	Deflection, mm Experiment	Crack width, mm (Experimental model)	Crack width, mm (Proposed formula)
80	1.2975	0.054	0.0223
150	2.456	0.094	0.0921
300	4.741	0.188	0.201
450	7.177	0.223	0.285
600	9.1455	0.265	0.338
800	11.8885	0.336	0.403
900	13.0965	0.349	0.432

**Figure A1:**
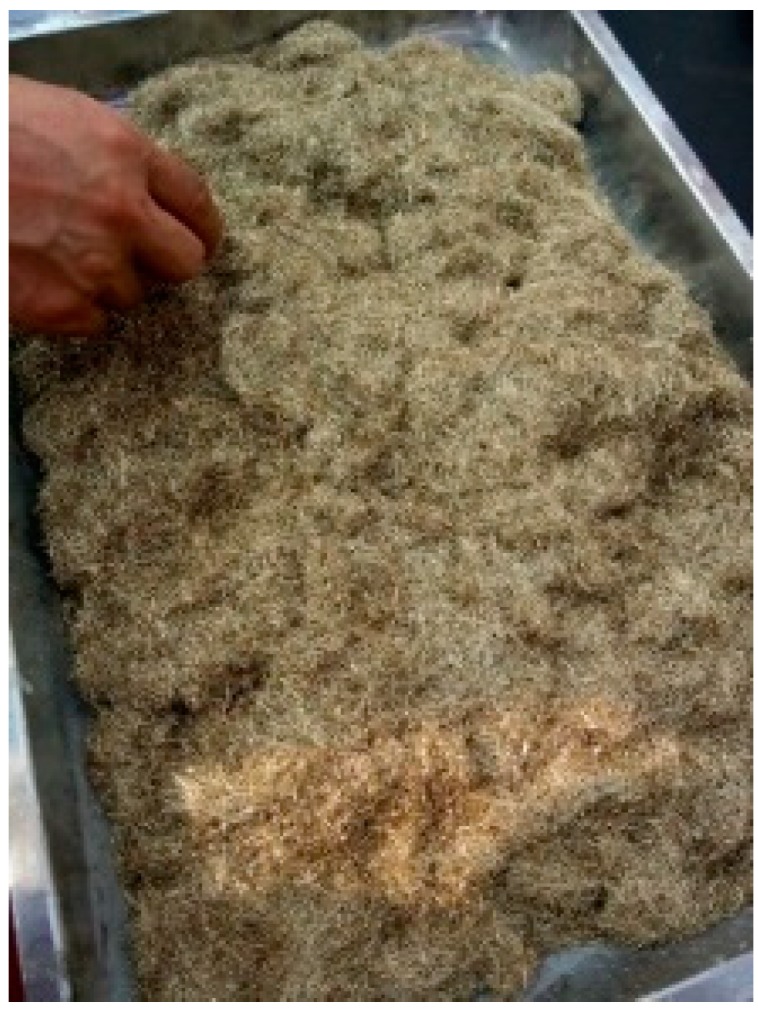
Steel fiber in concrete.

## Supplementary Materials

Supplementary File 1

## Notations

$\alpha_{t}$	parameter of concrete uniaxial tension stress–strain curve in the decline period
$f_{t,r}$	representation of concrete uniaxial tensile strength
$\varepsilon_{t,r}$	peak tensile strain corresponding to $f_{t,r}$
$d_{t}$	evolution parameter of concrete under uniaxial tension
$\alpha_{c}$	parameter of concrete uniaxial compression stress–strain curve in the decline period
$f_{c,r}$	representation of concrete uniaxial compressive strength
$d_{c}$	evolution parameter of concrete under uniaxial compression
$\varepsilon_{c,r}$	peak compressive strain corresponding to *f_c,r_*
*C*	crack width
*d*	deflection
*L*	applied load
Φ	diameter of rebar and stud
*t*	thickness of web and flange

## Appendix A

The cement was made of local brand of 52.5 grade OPC, and Ganjiang river medium sand was used for fine aggregate with a fineness modulus of 2.8; well-graded basalt was used for coarse aggregate with a maximum particle size less than 16mm. The main chemical composition for bonding properties of the constituent materials are shown in Table A1. The average fineness of the slag powder, density, water reduction rate, activity index on 28d, and activity index on 56d were 3 microns, 2.40 g/cm^3^, 10–15%, 105–110% on 28d, and 110–125%, respectively.

**Table A1:** Chemical constituents of Diatomite, SiO_2_ and mineral powder (%).

Component	SiO_2_	Al_2_O_3_	MgO	CaO	f-CaO	SO_3_	MnO	Density	Loss
Diatomite	76.11	11.21	3.5	3.8	-	-	-	2.6	<1
Sub-nano SiO_2_	80.11	1.49	2.69	4.64	1.98	0.65	-	-	<1.5
Micron grade Mineral powder	33.84	11.68	10.61	38.13	-	-	0.34	-	<3

Silicon Powder was made by local Building Materials Co., Ltd. according to GB/T176-1996 [55]. The test results of Silicon powder measured by GB/T18736/2002 [56] is reported in Table A2.

**Table A2:** Silicon powder performance index.

Item	SiO_2_ (%)	Moisture Content (%)	Ignition Loss (%)	Water Demand Ratio (%)	Fineness (45 um) (%)	28d Activity Index
Control index	≥85	≤3.0	≤6	≤125	-	≥85
Test Result	95.3	0.90	2.1	119.5	0.9	90

Steel fibers: Straight copper-plated steel fibers with a diameter of 0.2 mm, length of 13 mm, tensile strength of 2000 MPa, and volume fraction of 1.5% were used as steel fibers. Figure A1 shows the steel fibers mixed with C80 high-performance concrete. Table A3 shows the physical properties of the steel fibers utilized in the experimental test.

**Table A3:** Physical properties of steel fibers.

Fiber type	Length (mm)	Diameter (mm)	Aspect Ratio	Tensile Strength (MPa)	Modulus of Elasticity (GPa)	Density (Kg/m^3^)
Steel fiber	13	0.2	65	2000	210	7800

Water reducer: Superplasticizer of a local chemical company was used. The index parameters are listed in Table A4.

**Table A4:** Performance index of water reducer.

Item	PH Value	Density (g/ml)	Solid Content (%)	Chloride Ion Content (%)	Alkali Content (%)	1 h Loss	Water Reduction Rate.
Control Index	6.5 ± 0.02	1.04 ± 0.02	21±1	≤0.2	≤3.0	40	≥25
Test result	6.5	1.046	21.5	0.05	0.12	23	30.4

Water: Ordinary tap water was used in the experiment.

C60 concrete and C80 concrete were tested, and each concrete was designed with three mixing ratios. Three sets of test pieces were made for each combination, and each group had three cube test pieces. After conducting the strength test, the design mix ratio was optimized. The concrete mix of C60 and C80 is shown in Table A5.

**Table A5:** Benchmark mixture proportion of C60 and C80 high-performance concrete.

Concrete	Water Binder Ratio	Sand Rate (%)	The Amount of Raw Mterial Used Oer Concrete							
			Cement	Slag Powder	Fine Aggregate	Coarse Aggregate	Water	Water Reducer	Silica Fume	Steel Fiber
C60 *	0.3	40	388	129	699	1049	155	6.2	-	-
C80 **	0.25	38	435	87	652	1063	157	10.44	58	87

* Cement = 0.75, slag powder = 0.25, fine aggregate = 1.35, coarse aggregate = 2.03, water = 0.3, Admixture = 0.012. ** Cement = 0.75, slag powder = 0.15, Silica powder = 0.1, fine aggregate = 1.124, coarse aggregate = 1.83, steel fiber = 0.15, water = 0.27, and water reducer = 0.018.

When the C60 high-performance concrete bridge deck of the test beam was prefabricated, eight groups of 150 mm cubic test blocks and two groups of 150 × 150 × 300 mm prism test blocks were made. Four groups of cube blocks with the same curing conditions of the bridge deck were tested. For two groups of cubes, compressive strength was measured when the cumulative temperature reached 600 °C. One group was tested for cube compressive strength on the test day, and the other was preserved. The remaining four groups were them used for standardization; among them, two groups were used for measuring 7-day compressive strength, one group was used for measuring 28-day compressive strength of concrete cube, and one group was used for testing splitting strength. The prismatic test block is standard for testing the 28-day axial compressive strength of concrete and elastic modulus. Three groups of 150 mm cubic test blocks and two groups of 150 × 150 × 300 mm prismatic test blocks were made when C80 steel fiber high-performance concrete was poured into the reserved holes. Two groups of cubic specimens were used to obtain the 28-day cubic strength and splitting strength of C80 steel fiber high-performance concrete and the remaining one group was preserved. At the same time, two groups of prism specimens were used to test the 28-day axial compressive strength and elastic modulus of concrete. Both cube and prism specimens were loaded by universal testing machine. The test results and elastic modulus of C60 high-performance concrete cube under standard curing and actual curing condition (i.e., the same curing condition with bridge deck) are shown in Table A6, Table A7 and Table A8. The test results of C80 steel fiber high-performance concrete cube and elastic modulus are shown in Table A9 and Table A10.

**Table A6:** Concrete strength obtained from the experimental test on standard C60 HPC cubic specimen.

Test Block Number	Standardized 7-day Cube Strength (MPa)		Standardize 28-day Cube Strength (MPa)	Splitting Strength (MPa)
Test block 1	63.4	55.9	77.8	5.63
Test block 2	55.2	54.1	69.9	5.08
Test block 3	48.9	57.9	67.2	4.56
Average value	55.9		71.6	5.09

**Table A7:** Concrete strength obtained from experimental test on C60 HPC cubic specimen at the same condition with bridge deck.

Test Block Number	Compressive Strength (MPa) *		Strength Measured at the Test Day (MPa)
Test block 1	71.3	64.9	77.87
Test block 2	71.4	61.3	75.37
Test block 3	64.7	69.3	75.47
Average value	67.15		76.24

* Strength measured at the same curing condition with bridge deck.

**Table A8:** Elastic modulus obtained from the experimental test on C60 HPC.

Item	First Group	Second Group	Third Group	Average Value
Modulus of Elasticity (GPa)	36.0	36.7	36.5	36.4

**Table A9:** Concrete strength obtained from the experimental test on standard C80 HPC cubic specimen.

Test Block Number	Standard Curing 28-day Strength (MPa)	Splitting Strength (MPa)
Test block 1	90.4	8.27
Test block 2	87.5	9.91
Test block 3	84.4	8.83
Average value	87.4	9

**Table A10:** Elastic modulus and Poisson’s ratio obtained from the experimental test on C80 HPC.

Item	First Group	Second Group	Third Group	Average Value
Modulus of elasticity (GPa)	37.1	36.1	36.3	36.5
Poisson ratio	0.183	0.181	0.195	0.186
