# Supplementary figures and images for: A Semi-Empirical Deflection-Based Method for Crack Width Prediction in Accelerated Construction of Steel Fibrous High-Performance Composite Small Box Girder
Source: Materials (Basel). 2019 Mar 22;12(6):964. doi: 10.3390/ma12060964 (PMC6471295; doi:10.3390/ma12060964)

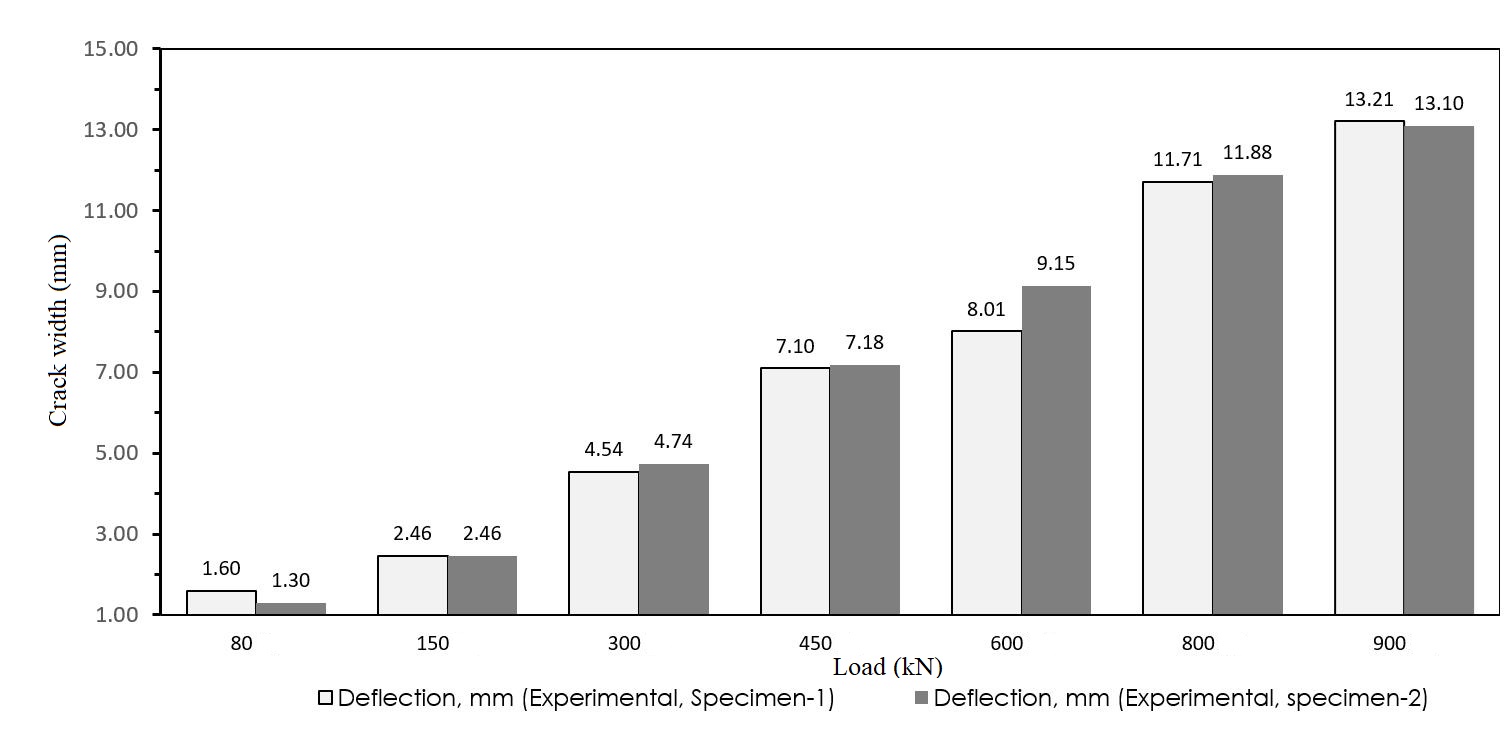

Supplement: Supplementary File 1 [file materials-12-00964-s001.zip › Fig. 14 and Fig. 18/Figure 14.jpg]

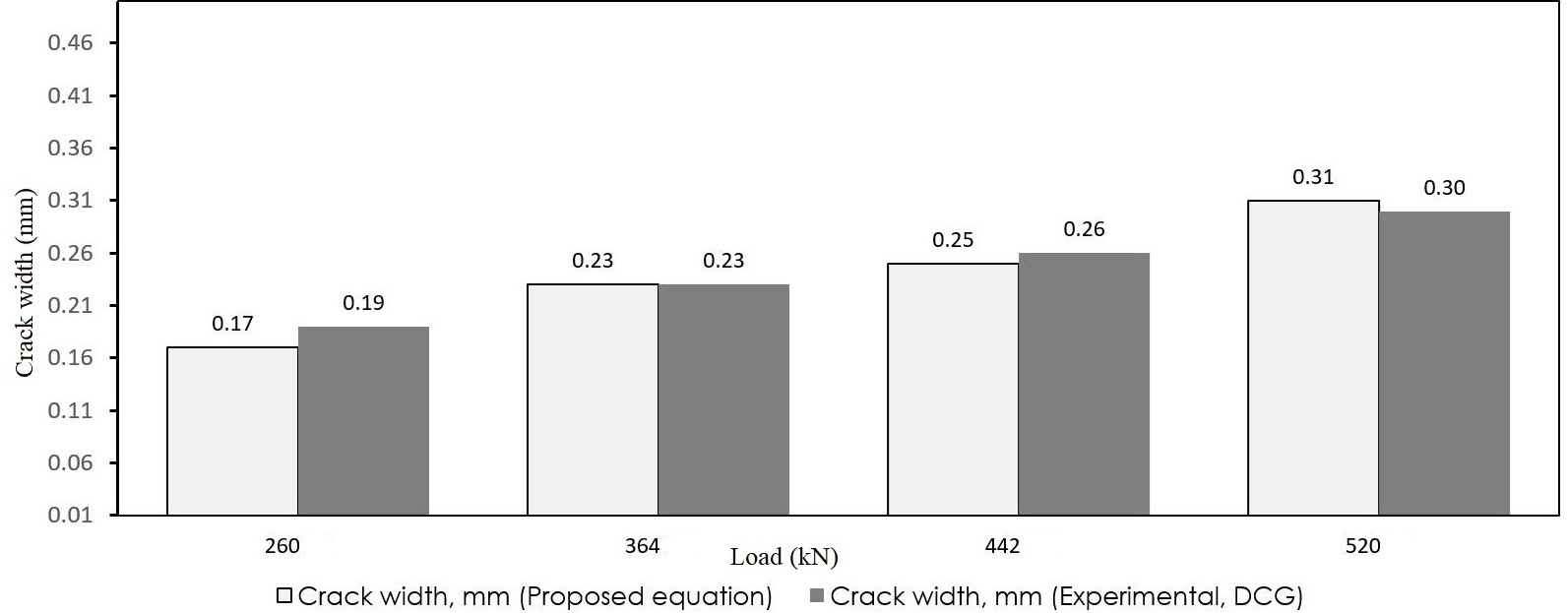

Supplement: Supplementary File 1 [file materials-12-00964-s001.zip › Fig. 14 and Fig. 18/Figure 18.jpg]
